# Plastic bezoar causing acute pancreatitis in an adolescent: A case report

**DOI:** 10.1097/PG9.0000000000000026

**Published:** 2020-12-17

**Authors:** Dominic Staudenmann, Arthur J Kaffes, Payal Saxena

**Affiliations:** From the * AW Morrow Gastroenterology and Liver Centre, Royal Prince Alfred Hospital, Sydney, NSW, Australia; † The University of Sydney, Sydney, Australia.

Children account for roughly 75% of patients with foreign bodies (FB) in the upper GI tract (1). FB are generally swallowed accidentally or purposefully. Their ingestion has extremely low morbidity and mortality rates, particularly after the object has passed the oesophagus. Gastrointestinal bezoars are collective of inedible or undigested material found in the GI tract, most commonly in the stomach and accounting for a significant proportion of FBs. Clinical symptoms vary from no symptoms to major complications including gastric ulcer, gastric perforation, and intestinal obstruction. Bezoar causing duodenal obstruction with secondary AP is extremely rare and only a few case reports have been published about trichobezoar (2, 3).

## CASE REPORT

A 16-year-old boy was admitted because of epigastric pain with ongoing nausea and vomiting for 1 week. The patient had no relevant past medical history. On physical examination, he appeared acutely ill, with a soft abdomen but with tenderness in the epigastric area and right upper quadrant. His laboratory tests revealed white blood cell count 10.7 10^9^/L (normal, 4–10 10^9^/L), aspartate aminotransferase level 168 U/L (normal, 0–40 U/L), alanine aminotransferase level 549 U/L (normal, 0–40 U/L), alkaline phosphatase level 494 U/L (normal, 40–250 U/L), total bilirubin level of 35 µmol/L (normal, 1.71–20.5 µmol/L), amylase level of 375 U/L (normal, 20–125 U/L), and lipase level of 1390 U/L (normal, 13–60 U/L).

**FIGURE 1.**
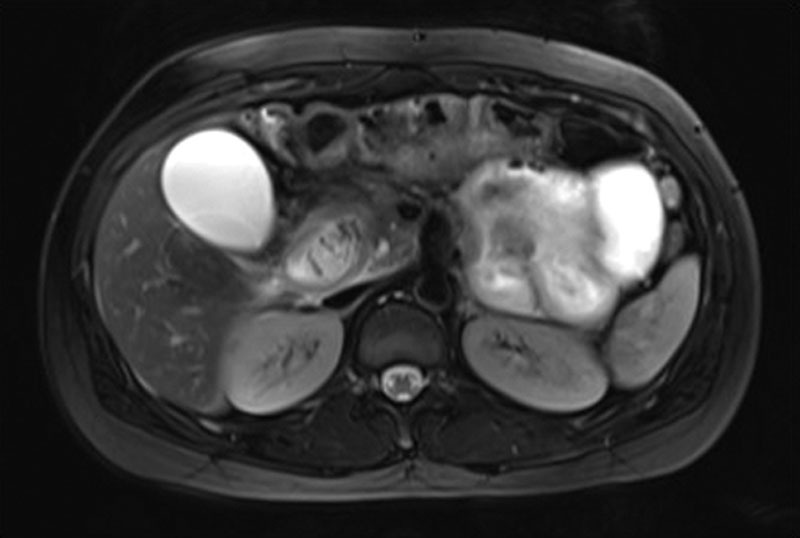
MRCP showed features of acute interstitial pancreatitis. MRCP, magnetic resonance cholangiopancreatography.

**FIGURE 2.**
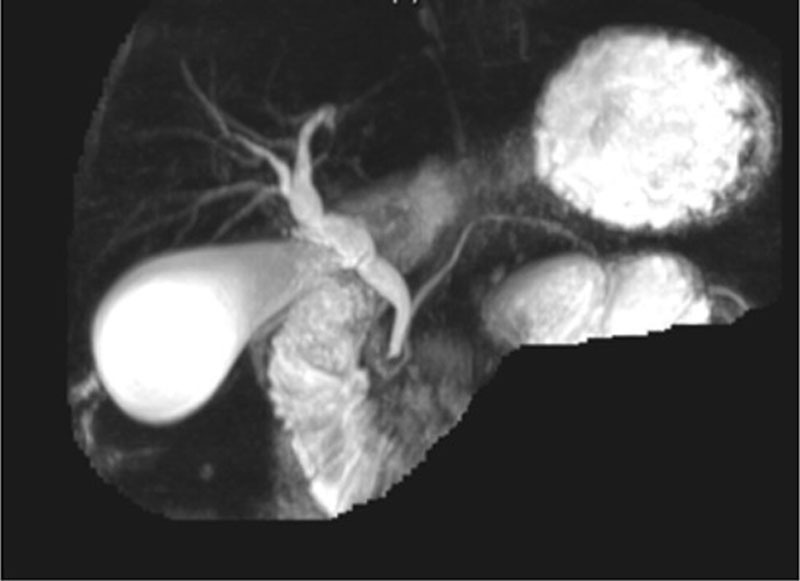
MRCP revealed a mild dilatation of the common bile duct (CBD) and hepatic ducts. CBD, common bile duct; MRCP, magnetic resonance cholangiopancreatography.

Magnetic resonance cholangiopancreatography showed features of acute interstitial pancreatitis (Figure 1) with mild dilatation of the common bile duct (CBD) and hepatic ducts (Figure 2). No definite cholelithiasis or choledocholithiasis was observed. The gallbladder appeared normal, and there was no evidence of pancreas divisum or other abnormality.

**FIGURE 3.**
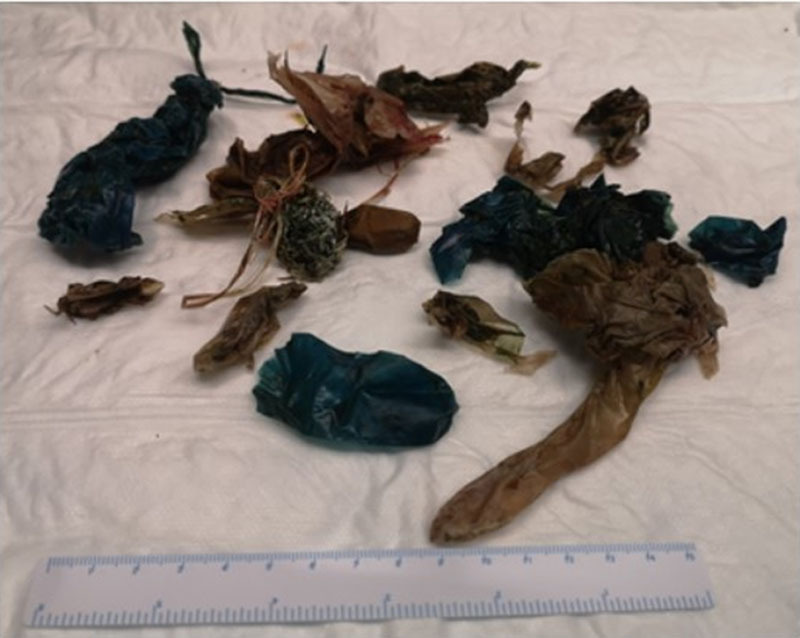
Plastic FBs up to 12-cm long. FB, foreign bodies.

Endoscopy ultrasound (EUS) was performed for suspected pancreatobiliary obstruction. On entering the stomach large plastic conglomerates were seen, making it impossible to advance the device into the duodenal bulb. The gastroscope revealed that the pylorus, pars 1 and pars 2 duodeni were almost completely obstructed with plastic bezoars. Over 15 plastic FBs up to 12 cm long were extracted perorally using snares and forceps (Figure 3). After extraction of the FBs, a 2-cm large ulcer was found at the papilla, and pus was emanating from the ampulla. Five days after admission, he was discharged from hospital without any gastric symptoms, continuing the antibiotic therapy (Ampicillin 2 g every 12 hours) for a total of 6 days. He admitted that he had been eating plastic parts regularly for 2 years, as part of coping strategies for distress management and grief. The young patient was seen by the mental health team in hospital and diagnosed with a Pica-syndrome (DSM-5 307.52 (F98.3)). According to the DSM-5 criteria, Pica is an eating disorder, and the diagnosis is given to someone who regularly and persistently eats non-food substances. The adolescent will be followed up with a psychiatrist.

## DISCUSSION

Acute pancreatitis (AP) in children and adolescents is not necessarily a rare disease; it is estimated that the annual incidence of paediatric AP is approaching that of adults at roughly 1/10,000 children per year (4, 5).

As with adults, paediatric AP is associated with significant disease burden. Children and adolescents with AP are hospitalized in average for 5–8 days, while infants and toddlers spend even up to 20 days in the hospital (6). Thus, AP should always be considered in the differential diagnosis of abdominal pain in children, and appropriate treatment should be started promptly.

The etiology of pancreatitis in children and adolescent has a different distribution than in adults. Obstructive factors, medications (in particular antiepileptics and immunosuppressors) and idiopathic origins are the main causes of AP in the paediatric age group accounting for approximately 75% of all the cases. Systemic diseases, trauma, viral infections (influenza, mumps, measles, coxsackie, Epstein bar virus, etc.), and others make up roughly 5%–10% each. First episodes of AP triggered by genetic mutations, metabolic factors or alcohol are not common in children (7).

Although FB ingestion is common in children, this hardly ever leads to AP. In adolescents the literature provides only a few cases of AP secondary to trichobezoars. It is often observed in young female patients with long hair, in many cases associated with the psychiatric disorders trichotillomania and trichophagia. Rapunzel syndrome is an extreme form of trichobezoar in which a part of the hair ball extends into the intestines. It is related to severe complications, one of them being AP (2, 8). Our patient had a unique plastic bezoar due to Pica syndrome. Although the abnormal eating disorder of plastikophagia has been reported earlier (9), associated AP has never been reported. The objective of the treatment is the mechanical removal of the bezoar and the prevention of recurrences with psychotherapy, like we have established in our case. Extraction of the mass can often be done endoscopically or if needed surgically with laparotomy or laparoscopy (8).

To the best of our knowledge, this is the first case of AP in an adolescent caused by compression of the papilla through an impacted plastic conglomerate. Although FBs are an uncommon cause of pancreatitis, they should be considered as a differential diagnosis. Furthermore, one must underscore the importance of duodenal endoscopy in AP, especially in cases with an unknown aetiology or if a FB has to be extracted. Like in our case, FBs are often radiolucent and thus cannot be seen on imaging.
